# Thermostabilization of the uronate dehydrogenase from *Agrobacterium tumefaciens* by semi-rational design

**DOI:** 10.1186/s13568-017-0405-2

**Published:** 2017-05-23

**Authors:** Teresa Roth, Barbara Beer, André Pick, Volker Sieber

**Affiliations:** 1 Chair of Chemistry of Biogenic Resources, Straubing Centre of Science, Technical University of Munich, Schulgasse 16, 94315 Straubing, Germany; 2 Roche Diagnostics GmbH, Nonnenwald 2, 82377 Penzberg, Germany; 3 TUM Catalysis Research Center, Ernst-Otto-Fischer-Straße 1, 85748 Garching, Germany; 4 Fraunhofer Institute for Interfacial Engineering and Biotechnology IGB, Bio, Electro and Chemocatalysis BioCat, Straubing Branch, Schulgasse 11a, 94315 Straubing, Germany

**Keywords:** Uronate dehydrogenase, Glucuronic acid, *Agrobacterium tumefaciens*, Thermostability, B factor, Neutral drift

## Abstract

Aldaric acids represent biobased ‘top value-added chemicals’ that have the potential to substitute petroleum-derived chemicals. Until today they are mostly produced from corresponding aldoses using strong chemical oxidizing agents. An environmentally friendly and more selective process could be achieved by using natural resources such as seaweed or pectin as raw material. These contain large amounts of uronic acids as major constituents such as glucuronic acid and galacturonic acid which can be converted into the corresponding aldaric acids via an enzyme-based oxidation using uronate dehydrogenase (Udh). The Udh from *Agrobacterium tumefaciens* (UdhAt) features the highest catalytic efficiency of all characterized Udhs using glucuronic acid as substrate (829 s^−1^ mM^−1^). Unfortunately, it suffers from poor thermostability. To overcome this limitation, we created more thermostable variants using semi-rational design. The amino acids for substitution were chosen according to the B factor in combination with four additional knowledge-based criteria. The triple variant A41P/H101Y/H236K showed higher kinetic and thermodynamic stability with a T_50_^15^ value of 62.2 °C (3.2 °C improvement) and a ∆∆G_U_ of 2.3 kJ/mol compared to wild type. Interestingly, it was only obtained when including a neutral mutation in the combination.

## Introduction

The biocatalytic conversion of sugars from biomass-derived waste offers a promising route for the biotechnological production of fuels, chemicals and materials (Andberg et al. 2012). Next to sugars also sugar derivatives constitute an important building block of natural fibers and are therefore readily available.

Glucaric and other aldaric acids are considered top-value added chemicals to be obtained from biomass and have the potential for various applications such as a building block for polymers and hyperbranched polyesters (Werpy and Petersen 2004). Currently glucaric acid is produced from glucose using strong oxidants like nitric acid (Werpy and Petersen 2004). This process is expensive and not selective. Higher selectivity could be achieved with an enzyme-based system for the production of glucaric acid. Moon et al. (2009) already constructed an enzyme-based pathway for the conversion of glucose to glucaric acid. The three enzymes *myo*-inositol-1-phosphate synthase, *myo*-inositol oxygenase and uronate dehydrogenase were recombinantly expressed in *E.* *coli.* However, only a yield of 17.4% (0.72 g/l) was achieved due to competition with the endogenous metabolism, which may limit carbon flux into the pathway for glucaric acid production. By inhibiting this flux through knockdown of phosphofructokinase an improvement up to a yield of 42% (1.56 g/l) could be achieved (Reizman et al. 2015). Due to the use of multiple enzymes this enzyme-based production of glucaric acid is still complex and insufficient. Instead of using glucose as the basic raw material the two ubiquitous sugar derivatives glucuronic acid and galacturonic acid would be more suitable to gain aldaric acids. Glucuronic acid is a component of hemicellulose, hyaluronic acid and seaweed, whereas the plant polysaccharide pectin largely consists of galacturonic acid (Ahn et al. 2012; Andberg et al. 2012). They can be oxidized to the corresponding aldaric acids by the enzyme uronate dehydrogenase (Udh, EC 1.1.1.203). For this conversion only the enzyme Udh would be required to obtain an enzyme-based system. The cell-based production of glucaric acid by Moon et al. (2009) is further limited by the intracellular accumulation of the product and the resulting acidification. This problem could be avoided by using a cell-free biosystem. A cell-free production has further advantages, i.e. easy control of the process, no substrate or product toxicity, higher product titer and broad reaction conditions (Guterl et al. 2012; You and Zhang 2012). Moreover the necessary cofactor nicotinamide adenine dinucleotide could easily be recycled to allow process operation under economic conditions. The recently characterized NADH oxidase from *Lactobacillus pentosus* which only forms water as byproduct would be a suitable enzyme in this process (Nowak et al. 2015).

The oxidation of uronic acids to aldaric acids by Udh was first described in the phythopathogenic bacteria *Pseudomonas syringae* and *Agrobacterium tumefaciens* (Zajic 1959). Until now several Udhs of the following organisms have been characterized: *Agrobacterium tumefaciens*, *Fulvimarina pelagi*, *Oceanicola granulosus*, *Streptomyces viridochromogenes*, *Pseudomonas syringae*, *Pseudomonas putida*, *Pseudomonas mendocina, Pseudomonas fluorescens, Polaromonas naphthalenivorans* and *Chromohalobacter salixigens* (Boer et al. 2010; Pick et al. 2015; Yoon et al. 2009; Wagschal et al. 2014). The best characterized enzyme is the Udh from *Agrobacterium tumefaciens* (UdhAt), which belongs to the short-chain dehydrogenase/reductase (SDR) superfamily and accepts only NAD^+^ as cofactor (Pick et al. 2015). The enzyme forms a hexamer in which two monomers interact tightly through the contact of the α-helices 3 and 4 (PDB code: 3RFT). Three dimers are then more loosely packed to form the hexamer. Each monomer consists of a single domain with the typical Rossmann fold for cofactor binding (Parkkinen et al. 2011; Pick et al. 2015). In comparison to the other Udhs the one derived from *Agrobacterium tumefaciens* features the highest catalytic efficiency (829 s^−1^mM^−1^) using glucuronic acid as substrate. Nevertheless, its short half-life of only 50 min at 37 °C is limiting its potential for industrial applications (Pick et al. 2015). Therefore the aim of this study was to overcome this limitation by improving the thermostability using enzyme engineering.

We chose a semi-rational design due to the availability of the crystal structure of the UdhAt. This helped to define positions for substitution, which could lead to a greater thermostability without losing or diminishing enzyme activity.

Systematic structural studies regarding mesophilic and thermophilic enzymes have shown that the latter are characterized by higher degrees of rigidity. This can be achieved by the accumulation of a variety of effects like hydrogen bonds or salt bridges. So, increasing the rigidity of the enzyme at appropriate sites should enhance thermostability (Reetz and Carballeira 2006). Therefore, the first and leading criterion was the B factor (atomic displacement parameter), which describes the movement of an atom around its mean position and therefore shows the flexibility and dynamics of the protein structure (Parthasarathy and Murthy 2000). The factor is determined for each atom in a protein structure during high-resolution X-ray crystallography. The average B factor for an amino acid position is then calculated by the program B-FITTER (Carballeira and Reetz 2007). Apart from this tool, informations on appropriate sites for mutagenesis can also be obtained from empirical studies that determine how amino acid side chains affect secondary or tertiary structures (Spector et al. 2000; Sriprapundh et al. 2000) or from looking at amino acid conservation (Anbar et al. 2012; Wijma et al. 2013). This rational approach for the selection of amino acids for mutagenesis is then combined with randomization using degenerated primers to find the best amino acid substitution at the specified positions. The generated smart enzyme libraries are small and can such easily be screened in a short period of time to find variants with improved thermostability.

## Materials and methods

### Reagents

All chemicals were of analytical grade or higher quality and purchased from Sigma-Aldrich, Molekula, Carl Roth, Alfa Aesar and VWR. For protein purification, equipment and columns, from GE Healthcare were used (Munich, Germany).

### Strains and plasmid

The strains *E. coli* XL1 BLUE and *E.* *coli* BL21(DE3) were used during this work. Construction of the plasmid pCBR-*udh*-*A.t.* was described by Pick et al. (2015).

### Enzyme expression and purification

*Escherichia coli* BL21(DE3) containing the plasmid of interest was grown in 250 ml autoinduction medium (Studier 2005). The preculture was incubated in 20 ml of LB medium with 30 µg/ml kanamycin at 37 °C overnight on a rotary shaker (180 rpm). The expression culture was then inoculated to reach an OD_600 nm_ of 0.1. Incubation was performed for 4 h at 37 °C followed by incubation for 21 h at 16 °C. Cells were harvested by centrifugation and resuspended in 50 mM potassium phosphate buffer (KPi) pH 8.0 (10 mM imidazol, 500 mM NaCl and 10% glycerol). Crude extracts were prepared with a Basic-Z Cell Disrupter (IUL Constant Systems) and subsequent incubation with MgCl_2_ (2.5 mM) and DNaseI (1 µg/ml) for 20 min at room temperature to degrade DNA. The insoluble fraction of the lysate was removed by centrifugation (20,000 rpm for 40 min at 4 °C). The supernatant was applied to an IMAC affinity column, 5 ml HisTrap™ FF, equilibrated with the resuspension buffer using the ÄKTA Purifier-system. The column was washed with 20 ml of resuspension buffer and the enzyme was eluted with 50 mM KPi buffer pH 8.0 (500 mM imidazol, 500 mM NaCl and 10% glycerol). Elution was monitored by UV (280 nm) and fractions containing protein were subjected to 12% SDS-Page described by Laemmli (1970). The molecular weight of UdhAt was calculated to be 31.21 kDa (including the additional amino acids of the N-terminal His_6_-tag) using the ProtParam tool (Expasy). Factions containing the eluted target protein were pooled and desalted using a HiPrep™ 26/10 Desalting column which was preliminary equilibrated with 50 mM ammonium bicarbonate pH 7.9. Protein concentrations were determined using a NanoPhotometer (IMPLEN) with 50 mM ammonium bicarbonate pH 7.9 as the reference and an extinction coefficient of 37,930 M^−1^cm^−1^ (ProtParam, Expasy).

### Enzyme assay

The Udh activity was determined photometrically by monitoring the increase of NADH at 340 nm with a Multiskan spectrum spectrophotometer (Thermo Fisher Scientific). The reaction mixture contained 25 mM KPi buffer pH 8.0, 1 mM NAD^+^, 5 mM MgCl_2_ and 10 mM glucuronic acid. Measurements were performed at 25 °C after adding 20 µl of purified enzyme (9.1 × 10^−5^ mg/ml). One unit of enzyme activity was defined as the amount of protein that oxidizes 1 µmol of NADH/min at 25 °C.

### Mutagenesis

Saturation mutagenesis libraries were generated using the QuikChange^®^ mutagenesis strategy from Stratagene (USA). The degenerated primers used (Table 1) contained the codon NNK. High quality of the generated libraries was verified by sequencing five clones per library (GATC Biotech, Cologne, Germany).

**Table 1:** Primers used for saturation mutagenesis

	Sequence 5′–3′
L38fw	GATCTGTCTCCG**NNK**GATCCGGCTGGTCCGAATGAAG
L38rv	CAGCCGGATC**MNN**CGGAGACAGATCTGCCAGAC
A41fw	CCGCTGGATCCG**NNK**GGTCCGAATGAAGAATGTGTTC
A41rv	CTTCATTCGGACC**MNN**CGGATCCAGCGGAGACAGATC
E81fw	GCGTTGAAAAACCGTTT**NNK**CAGATTCTGCAGGGTAACATTATTGGC
E81rv	CCCTGCAGAATCTG**MNN**AAACGGTTTTTCAACGCTAATGCCAC
H101fw	GCAGCACGTGCA**NNK**GGTCAGCCTCGTATTGTTTTTGCAAG
H101rv	CAATACGAGGCTGACC**MNN**TGCACGTGCTGCTTCATACAG
H236fw	GCCTTTCGTCGT**NNK**ATTACCGAAACCACACCGCCTCCG
H236rv	GGTGTGGTTTCGGTAAT**MNN**ACGACGAAAGGCTTCTGCATTATCTTTCGG
E239fw	GTCGTCATATTACC**NNK**ACCACACCGCCTCCGGATCCGAATG
E239rv	CCGGAGGCGGTGTGGT**MNN**GGTAATATGACGACGAAAGGCTTC

### Culture conditions for 96 deep-well plates

*Escherichia coli* BL21(DE3) containing the plasmid (plasmid libraries) of interest were used for expression in 96 well format. The colonies were picked using the Hudson Rapid Pick lite colony picker (Hudson Robotics Inc., Springfield, USA) and grown in 96 deep-well plates containing 1200 µl autoinduction medium (Studier 2005) with 100 µg/ml kanamycin, for 25 h at 37 °C on a rotary shaker (1000 rpm). 100 µl of the cultures were centrifuged (3000 rpm for 15 min at 4 °C), the supernatants discarded and the cell pellets frozen at −80 °C for at least 2 h. Afterwards 100 µl of 25 mM KPi pH 8.0 were added and the plates incubated for 1 h at 37 °C on a rotary shaker (700 rpm) for cell disruption.

### Screening

The screening for thermostability was assessed based on the residual activity subsequent to the exposure to high temperatures. The supernatants were diluted (1:2000 in a total volume of 50 µl) in incubation mixture containing 25 mM KPi pH 8.0, 5 mM MgCl_2_ and 100 mM glucuronic acid. Before incubation the initial activity was measured using an aliquot of 20 µl and adding 180 µl of reaction mixture (25 mM KPi pH 8.0 and 1 mM NAD^+^). Heat treatment was performed for 15 min at 58 °C in a PCR thermocycler. After cooling to 4 °C another aliquot of 20 µl was used to measure the residual activity. Variants showing a residual activity greater than the wildtype enzyme plus standard deviation were considered as hits.

### Kinetic stability

Kinetic stability can be described by T_50_^15^, the temperature at which 50% of the enzyme’s initial activity is left after incubation for a defined time period. For this purpose, a gradient PCR thermocycler was used. The purified enzymes were incubated at 50–64 °C at the same enzyme concentration (9.1 × 10^−5^ mg/ml) with 25 mM KPi pH 8.0, 5 mM MgCl_2_, 0.1 mg/ml BSA and 100 mM glucuronic acid.

### Thermodynamic stability

The thermodynamic stability was determined by guanidine hydrochloride (GdmCl) induced unfolding. Therefore, 100 µl protein were incubated with various concentrations of GdmCl (0–3.5 M) in 25 mM KPi pH 8.0 for 8 days at RT. The proteins were transferred into a 96-well optical-bottom plate (Thermo Fisher Scientific) and the fluorescence emission at 344 nm was measured after excitation at 278 nm in a Varioskan (Thermo Fisher Scientific). The difference in free energy of unfolding of WT and the variants (∆∆G_U_) was calculated using the following equation: $\Delta \Delta G_{U} = 0.5\,(m_{wild\,type} + m_{variant} )\Delta [GdmCl]_{50\% }$, where m is the slope of the linear denaturation plot −dAGu/d[denaturant] and Δ[GdmCl]_50%_ is the difference between [GdmCl]_50%_ for wild type and mutant (Kellis et al. 1989).

### Determination of kinetic parameters

Kinetic parameters (k_cat_ and K_M_) were determined for WT and purified variants. Measurements were performed in 25 mM KPi pH 8.0 at 25 °C with varying concentrations of glucuronate (0–10, 1 mM NAD^+^) or NAD^+^ (0–4, 10 mM glucuronate). The increase of NADH was monitored at 340 nm with a Multiskan spectrum spectrophotometer (Thermo Fisher Scientific). The data was fitted to the Michaelis–Menten equation using SigmaPlot 11.0.

## Results

### Identification of amino acid positions for mutagenesis

Site-saturation mutagenesis has proven to be a useful strategy to alter enzyme properties like thermal stability or substrate specificity when the amino acid positions are properly selected (Reetz and Carballeira 2006). Setting the B factor as a criterion was already suggested by Parthasarathy and Murthy (2000) and further successfully applied for thermal stabilization of *Bacillus subtilis* lipase (Reetz and Carballeira 2006) and an α-Amino ester hydrolase (Blum et al. 2012). Hence, we used the B factor as our leading criterion. In multiple studies the B factor was combined with the structure-guided consensus method to reduce the number of amino acids to be mutated (Blum et al. 2012; Jochens et al. 2010). In our case a combination of those methods was not suitable because the consensus sequence of all known Udhs (using the recommended cut off of 80%) was identical to the sequence of the UdhAt. Therefore, we chose four other criteria in combination with a B factor greater than 25 Å^2^: first, the localization of amino acids within the protein was considered. Amino acids within elements of pronounced secondary structure were excluded for mutagenesis except when they have a low propensity for this type of secondary structure (Bommarius and Paye 2013; Lehmann and Wyss 2001). In addition amino acids that are within the cofactor and substrate binding sites or at the interface between the protein domains of the multimer (α-helices 3 and 4) were not considered. Second, amino acids that occur more often in proteins from thermophilic origin and are typically considered for protein stabilization such as proline, arginine and tyrosine were excluded from mutagenesis (Querol et al. 1996; Lehmann and Wyss 2001). Furthermore, conserved amino acids as well as amino acids that are probably involved in a hydrogen bonding network (PyMOL) were excluded. Conserved amino acids are advantageous for the protein and “survived” during evolution (survival of the fittest) (Bommarius and Paye 2013). Therefore, sequence and structure alignments of all known Udhs and enzymes that had a sequence similarity greater than 60% compared to the UdhAt were created. From these alignments consensus sequences with coverage of 95 or 80% were created (BioEdit and PROMALS) and all amino acids that showed conservation were excluded from mutagenesis. An overview of these five criteria and the amino acids considered for mutagenesis is shown in Table 2. Six amino acids fulfilled all five criteria: L38, A41, E81, H101, H236 and E239. They were subjected to site-directed mutagenesis via QuikChange PCR using degenerated primers with an NNK motif, covering at least one codon of all canonical amino acids.

**Table 2:** The five criteria for selecting the amino acid positions for mutagenesis

Criterion	Amino acids
B factor >25 Å^2^	K4, Q14, R17, E21, A24, P25, M26, E28, S36, P37, L38, D39, P40, A41, G42, P43, N44, E45, E46, Q49, A63, P79, E81, H101, G134, F154, C166, T167, P168, E169, N171, F180, S181, E190, H218, G223, K227, R235, H236, T238, E239, T240, T241, P242, P243, P244
Location	K4, M26, P37, L38, D39, P40, A41, G42, P43, N44, E45, A63, P79, E81, H101, T167, P168, E169, N171, F180, E190, K227, H236, T238, E239, P242, P243, P244
Occurrence in thermostable proteins	K4, M26, L38, D39, A41, G42, N44, E45, A63, E81, H101, T167, E169, N171, F180, E190, K227, H236, T238, E239
Conservation	M26, L38, D39, A41, G42, N44, A63, E81, H101, E169, N171, F180, E190, K227, H236, T238, E239
Part of hydrogen bonding network	L38, A41, E81, H101, H236, E239

### Screening of mutant libraries

The stability of proteins can be judged by three types of criteria: kinetic, thermodynamic and process stability (Bommarius and Paye 2013).

Fast screening to examine improved thermostability in the initial six libraries was performed in 96-well PCR plates by heating the enzyme solutions (diluted supernatants) to 58 °C for 15 min in a thermocycler. The temperature of 58 °C was chosen because it reduced WT activity to 10% (standard deviation of 2%) allowing a fast identification of positive hits. In library H236 three variants showed a higher stability: H236K, H236I and H236R. All other enzyme variants in all other libraries showed lower thermostability than WT with exception of the variants A41P, H101Y and H101N, which were as stable as wild type enzyme. With only one position giving rise to improvements no combination of improved variants for possible additive or even synergistic effects was possible. However, recently several interesting studies had shown the importance of neutral drift on the evolution of enzymes (Gupta and Tawfik 2008; Smith et al. 2011). This led us to combine the mutations that were positive in the screen with the ones that at least did not show any decrease in activity. We created a series of double and triple variants in addition to the single variants H236K, H236I and H236R (see Table 3).

**Table 3:** Created single, double and triple variants of UdhAt to test for additive or synergistic effects

Single variants	Double variants, combining single variants and:			Triple variants, combining single variants and:	
	A41P	H101Y	H101N	A41P/H101Y	A41P/H101N
H236K	A41P/H236K	H101Y/H236K	H101N/H236K	A41P/H101Y/H236K	A41P/H101N/H236K
H236I	A41P/H236I	H101Y/H236I	H101N/H236I	A41P/H101Y/H236I	A41P/H101N/H236I
H236R	A41P/H236R	H101Y/H236R	H101N/H236R	A41P/H101Y/H236R	A41P/H101N/H236R

Again, the residual activity after incubation at 58 °C was measured (data not shown). Only the double variant A41P/H236R showed a reduced thermostability in the screen. The remaining 17 variants were purified and their kinetic and thermodynamic stability was compared as well as their kinetic parameters determined.

### Kinetic stability

**Fig. 1:**
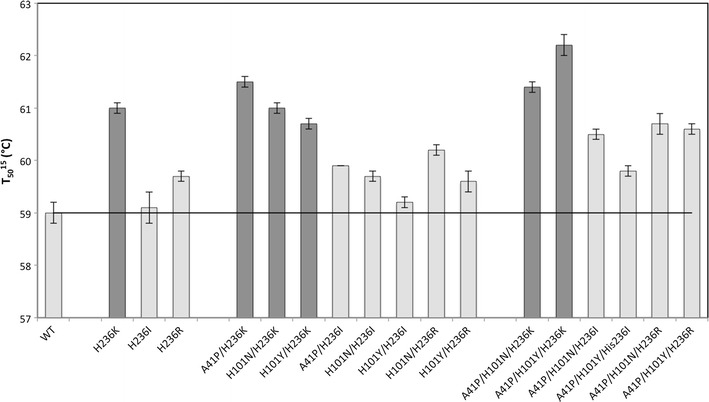
T_50_^15^ value of WT and 17 variants of UdhAt. Variants containing the mutation H236K are marked *dark grey*. The *solid line* indicates the T_50_^15^ value of WT (59 °C).* Error bars* are the standard deviation of four independent measurements

The T_50_^15^ value was defined as the temperature required to reduce the initial enzyme activity to 50% within 15 min. The T_50_^15^ of the purified WT enzyme was 59.0 °C. In Fig. 1 the T_50_^15^ of WT and variants are shown. All variants had an improved or equal kinetic stability compared to WT. The highest improvement of kinetic stability was observed for the triple variant A41P/H101Y/H236 K with a T_50_^15^ value of 62.2 °C, resulting in a ∆T_50_^15^ of 3.2 °C compared to WT.

### Thermodynamic stability

**Fig. 2:**
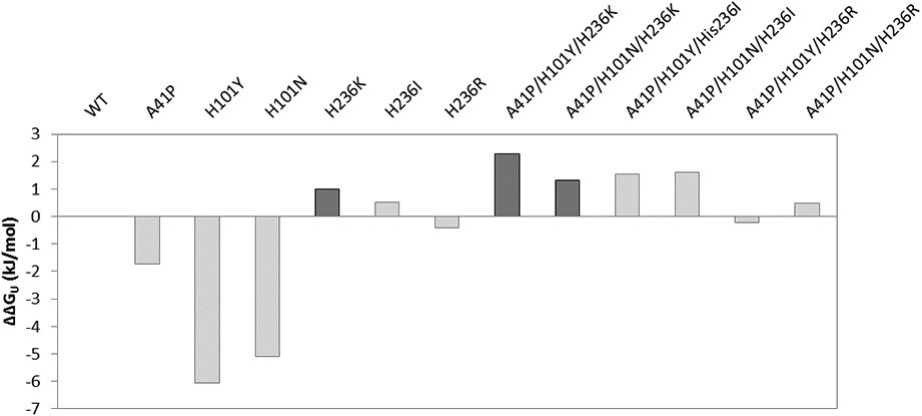
Differential thermodynamic stability of single and triple variants of UdhAt in comparison to WT using GdmCl as denaturing agent. The single variant and all other variants containing the mutation H236K are marked *dark grey*

**Fig. 3:**
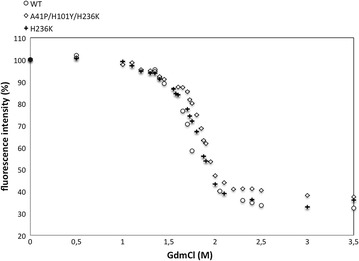
Fluorescence intensity of WT, the best triple variant A41P/H101Y/H236K and the single variant H236K. All data points are the mean value of twenty samples with a standard deviation of <0.5

The thermodynamic stability was determined by GdmCl-induced protein unfolding. The differences in free energy of unfolding of the single and triple variants compared to WT (∆∆G_U_) are shown in Fig. 2. The single variants all showed negative ∆∆GU compared to the wildtype, except H236K and H236I. However, the ∆∆GU of all double and triple variants—with exception of A41P/H101Y/H236R—were positive, which indicates that these variants have a higher thermodynamic stability than WT. Moreover, this stabilization is non-additive, as the single variants that were neutral in the screening, showed even lower stability than the WT when tested in the purified form. The best variant was the triple variant A41P/H101Y/H236K with a ∆∆G_U_ of 2.3 kJ/mol. In Fig. 3 the unfolding curves of WT, the single variant H236K and the best variant A41P/H101Y/H236K are shown.

### Kinetic parameters

The kinetic parameters v_max_, K_m_ and k_cat_/K_m_ for the substrate glucuronic acid and the cofactor NAD^+^ were determined for the eight best variants. WT had a specific activity of 390 U/mg, a K_m_ of 0.71 mM and a catalytic efficiency of 287 s^−1^mM^−1^. In comparison, the variants had a slightly reduced specific activity and a higher K_m_ value (Table 4) resulting also in a reduced catalytic efficiency (40–60% compared to WT).

**Table 4:** Kinetic parameters of the eight best variants and WT for glucuronic acid including the standard deviation of three measurements

Variant	Specific activity (U/mg)	K_m_ (mM)	Catalytic efficiency	
			(s^−1^mM^−1^)	(%)
WT	391 ± 17	0.7 ± 0.1	286.4 ± 13.0	100
H236K	234 ± 5	1.3 ± 0.1	97.5 ± 2.2	34.0
A41P/H236K	407 ± 8	1.2 ± 0.0	179.5 ± 3.5	62.7
H101Y/H236K	218 ± 6	1.0 ± 0.1	116.7 ± 3.4	40.8
H101N/H236K	228 ± 5	0.9 ± 0.1	139.7 ± 3.2	48.8
A41P/H101Y/H236K	280 ± 6	0.9 ± 0.1	167.3 ± 3.6	58.4
A41P/H101Y/H236R	302 ± 4	1.0 ± 0.1	160.5 ± 2.0	56.0
A41P/H101N/H236K	248 ± 4	0.9 ± 0.1	149.7 ± 2.25	52.3
A41P/H101N/H236R	330 ± 3	1.0 ± 0.0	170.0 ± 1.7	59.3

## Discussion

With the enzyme uronate dehydrogenase a selective one-step enzyme-based production of aldaric acids from waste biomass could be possible. The uronate dehydrogenase from *Agrobacterium tumefaciens* (UdhAt) features the highest efficiency among all known Udhs using glucuronic acid as substrate. However, the enzyme lacks stability with only a half-life of 50 min at 37 °C (Pick et al. 2015). This limits its potential for industrial application. As no Udh is known from thermophilic organisms, we developed more thermostable variants by enzyme engineering.

**Fig. 4:**
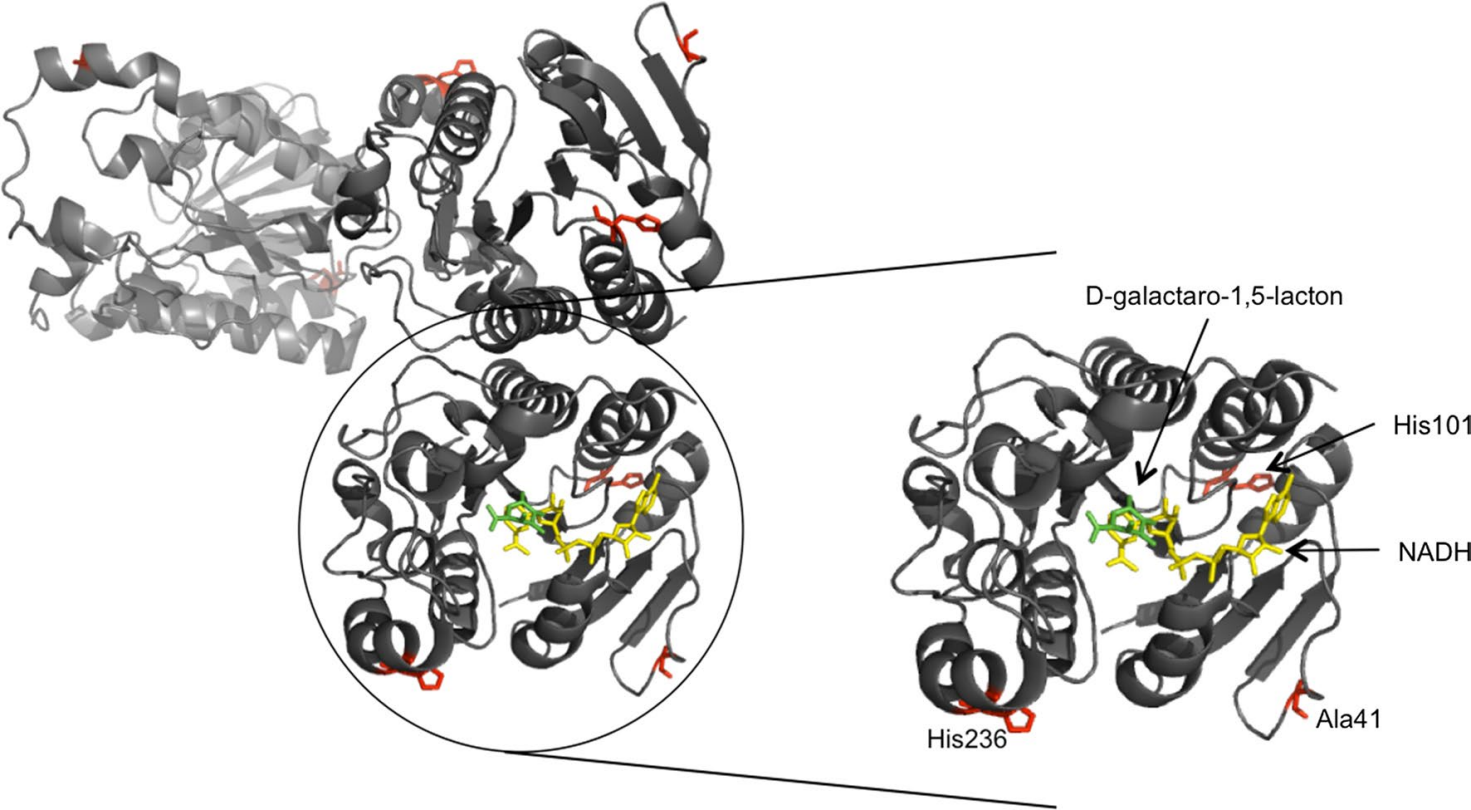
Three monomers of the UdhAt with a close-up of one highlighted with the mutated amino acids in *red*, the product d-galactaro-1,5-lactone in *green* and the cofactor NADH in *yellow*

This was achieved through the combination of an effective selection method for the amino acid positions to be mutated and the accumulation of advantageous mutations. The selection method for the amino acids was based on the B factor and four further criteria leading to the positions: L38, A41, E81, H101, H236 and E239. In the library H236 the three variants H236K, H236I and H236R had a greater thermostability than WT. The variants A41P, H101Y and H101N showed no change (positive or negative) and were therefore used to test for additivity. The triple variant A41P/H101Y/H236K showed the highest kinetic (∆T_50_^15^ = 3.2 °C) and thermodynamic stability (∆∆G_U_ = 2.3 kJ/mol) compared to WT. When two or more point mutations are introduced, the question arises whether they interact additively or non-additively. In the latter case they can cause either cooperative (positive) or antagonistic (negative) effects. It was suggested (Reetz 2013; Skinner and Terwilliger 1996) that additive effects might occur when the locations of mutations are well-separated. Whereas when the side chains of two residues are in close contact with one another their effects are generally non-additive. Our best variant A41P/H101Y/H236K showed non-additive synergistic cooperative effects, because the increase in thermostability was greater than the sum of the three single variants although the positions are not in close proximity (>20 Å^2^). Istomin et al. (2008) have obtained new insights concerning this topic. They concluded that a statistically significant bias toward non-additivity occurs whenever the residues, although not in direct contact, are located within the same rigid cluster. Additivity can be expected when they are in different clusters. Also Reetz et al. (2009) could show that the hyperthermophilic mutant XI of the lipase from *Bacillus subtilis* had cooperative non-additive effects between five distal residues. The stabilization was performed by the formation of an extensive H-bond/salt-bridge network on the surface of the enzyme. This could be the same case here because all three amino acid positions A41, H101 and H236 are located on the surface of the enzyme (Fig. 4). Furthermore, the substitution A41P helped to regain the catalytic function of the H236 mutation. The catalytic efficiency of the single variant H236K decreased to 34% of the wildtype activity, but was reconstituted to 63% in the double variant A41P/H236K. The triple variant A41P/H101Y/H236K with the highest stability still showed 56% of the wildtype activity (Table 4). This finding nicely demonstrates the potential of neutral drift mutations that have gained interest in the last decade (Bershtein et al. 2008; Bloom and Arnold 2009; Smith et al. 2011).

The mutation H236K had the greatest influence on thermostability of the UdhAt. The amino acid histidine at the position 236 had a high B factor (31.53 Å^2^) and was located in an α-helix. Histidine is not a good helix builder whereas the introduced amino acid lysine is. Interestingly, when we applied the strategy of Blum et al. (2012), who showed that consensus sequences with a rather low cut-off could lead to thermally more stable enzyme variants, Lysine 236 was indeed conserved (at a cut-off of 30%). In this consensus sequence 64 amino acids not identical to the UdhAt were conserved, i.e. E239A. Our screening assay did not find a preferable amino acid exchange at this position. At the positions A41, E81 and H101 wild type amino acid of UdhAt was conserved. This confirms the strategy of Blum et al. (2012) combining the B factor with the structure-guided consensus (SGC) concept: they chose WT positions that were not consensus and did not fit a number of structural guidelines and also amino acid positions with a high B factor and replaced them by the consensus amino acid. Thereby they could improve the thermostability of α-amino ester hydrolase by 7 °C. This strategy has also the advantage of small libraries and therefore less screening effort. Our strategy of choosing the amino acid positions for mutagenesis was also successful. Furthermore, we found the triple variant A41P/H101Y/H236K showing better thermostability than WT, which would not be detected by the SGC. It would be interesting to know if both methods–structure guided consensus concept with a low cut-off of 50% and applying our five criteria—would lead to the same results for thermostabilization when transferred to another Udh. For this purpose the Udh of *Chromohalobacter salexigens* would be suitable because a crystal structure is already available (Ahn et al. 2012).

In summary, we have shown that the combination of the B factor with knowledge and structure-based criteria is successful for generating thermostable proteins of the UdhAt. The best UdhAt triple variant (A41P/H101Y/H236K) showed an improved T_50_^15^ value of 3.2 °C and a higher thermodynamic stability (∆∆G_U_ = 2.3 kJ/mol). With this approach for improving the stability the UdhAt has been made available for biotechnological applications i.e. for the cell-free production of glucaric or galactaric acid.

## Abbreviations

Udh: uronate dehydrogenase
UdhAt: uronate dehydrogenase from *Agrobacterium tumefaciens*
SDR: short-chain dehydrogenase/reductase
WT: wild type
SGC: structure-guided consensus
*E. coli*: *Escherichia coli*
KPi: potassium phosphate
GdmCL: guanidine hydrochloride
